# Effect of two erosive protocols using acidic beverages on the shear bond strength of orthodontic brackets to bovine enamel

**DOI:** 10.1590/2177-6709.23.6.064-072.oar

**Published:** 2018

**Authors:** Catielma Nascimento Santos, Felipe de Souza Matos, Sigmar de Mello Rode, Paulo Francisco Cesar, Flávia Pardo Salata Nahsan, Luiz Renato Paranhos

**Affiliations:** 1 Universidade Federal de Sergipe, Programa de Pós-graduação em Odontologia (Aracaju/SE, Brazil).; 2 Universidade Estadual Paulista, Instituto de Ciência e Tecnologia, Programa de Pós-Graduação em Odontologia Restauradora (São José dos Campos/SP, Brazil).; 3 Universidade Estadual Paulista, Instituto de Ciência e Tecnologia, Departamento de Materiais Dentários e Prótese (São José dos Campos/SP, Brazil).; 4 Universidade de São Paulo, Faculdade de Odontologia, Departamento de Biomateriais e Biologia Oral (São Paulo/SP, Brazil).; 5 Universidade Federal de Sergipe, Departamento de Odontologia (Lagarto/SE, Brazil).; 6 Universidade Federal de Uberlândia, Faculdade de Odontologia (Uberlândia/MG, Brazil).

**Keywords:** Erosion, Orthodontics, Orthodontic brackets, Shear strength.

## Abstract

**Objective:**

To assess the short-term effect of two *in vitro* erosive challenge protocols on the bond strength of metal orthodontic brackets on bovine enamel.

**Methods:**

Sixty bovine incisors were selected and randomly divided into six groups: AS7 (artificial saliva - 7 days, Control Group); CC7 (Coca-Cola™ - 7 days); LJ7 (lime juice - 7 days); AS30 (artificial saliva - 30 days, Control Group); CC30 (Coca-Cola™ - 30 days); LJ30 (lime juice - 30 days). Microhardness testing was performed prior to the erosive challenge to verify the standardization of samples. Immersion was performed 4x/day for five minutes, for either 7 or 30 days. After immersions were concluded, the brackets were bonded and shear bond strength was assessed after 48 hours. The Adhesive Remnant Index (ARI) was also assessed. Data were analyzed by two-way ANOVA, followed by Tukey’s *post-hoc* and Student’s *t* test for paired samples, and the Kruskal-Wallis non-parametric test (α = 5%).

**Results:**

The mean and standard deviation of microhardness testing of total samples were 281.89 ± 44.51 KHN. There was no statistically significant difference in shear bond strength for the time factor (7 or 30 days; F_5.54_= 0.105; *p*_ _= 0.901). However, there was a statistically significant difference for the solution factor (F_5.54_= 6.671; *p*_ _= 0.003). These differences occurred among solutions of Saliva x Coca-Cola™ (*p*_ _= 0.003) and Coca-Cola™ x Lime Juice (p_ _= 0.029). The assessment of the Adhesive Remnant Index showed no significant difference between groups.

**Conclusions:**

The immersion time used in the erosion protocols did not affect the bond strength of brackets to teeth. Coca-Cola™ induced significantly higher shear bond strength values than lime juice and artificial saliva. However, the short term effects of 7/30 days in this *in vitro* study may not be extrapolated for *in vivo* ones. Clinical studies should be conducted, substantiating the laboratory results.

## Resumo

**Objetivo:**

avaliar o efeito de curto prazo de dois protocolos de desafio erosivo, *in vitro*, na resistência adesiva de braquetes ortodônticos metálicos em esmalte bovino.

**Métodos:**

Sessenta incisivos bovinos foram selecionados e divididos aleatoriamente em seis grupos: SA7 (saliva artificial - 7 dias, Grupo Controle); CC7 (Coca-Cola^®^ - 7 dias); SL7 (suco de limão - 7 dias); SA30 (saliva artificial - 30 dias, Grupo Controle); CC30 (Coca-Cola^®^ - 30 dias); SL30 (suco de limão - 30 dias). Foi realizado o teste de microdureza antes do desafio erosivo, para verificar a padronização das amostras. A imersão foi realizada quatro vezes ao dia, por cinco minutos, durante 7 ou 30 dias. Finalizadas as imersões, os braquetes foram colados e, após 48 horas, foi avaliada a resistência ao cisalhamento. O Índice de Adesivo Remanescente (IAR) também foi avaliado. Para análise dos dados, foram utilizados os testes ANOVA dois fatores, seguido do *post-hoc* de Tukey e teste *t* de Student para amostras pareadas, e o teste não-paramétrico de Kruskal-Wallis (α?#8197;= 5%).

**Resultados:**

a média e o desvio-padrão do teste de microdureza das amostras totais foi igual a 281,89 ± 44,51 KHN. Não houve diferença estatisticamente significativa na resistência ao cisalhamento para o fator tempo (7 ou 30 dias; F_5,54_= 0,105; *p*= 0,901). Contudo, houve diferença estatisticamente significativa para o fator solução (F_5,54_=6,671; *p*= 0,003). Essas diferenças ocorreram entre as soluções de Saliva *x* Coca-Cola^®^ (*p*= 0,003) e Coca-Cola^®^ *x* suco de limão (*p*= 0,029). Ao avaliar o Índice de Adesivo Remanescente, não foi possível verificar diferença significativa entre os grupos.

**Conclusões:**

o tempo de imersão utilizado nos protocolos de erosão não afetou a resistência de união dos braquetes aos dentes. A Coca-Cola^®^ induziu valores de resistência ao cisalhamento significativamente mais altos do que o suco de limão e a saliva artificial. No entanto, os efeitos em curto prazo de 7 e 30 dias, nesse estudo *in vitro,* não podem ser extrapolados para os estudos *in vivo*. Estudos clínicos devem ser conduzidos, fundamentando os resultados laboratoriais.

## INTRODUCTION

Dental erosion is a problem with increasing incidence in the worldwide population.^1^ This type of dental lesion is characterized by wear on the tooth surface caused by a chemical process involving the activity of acids, without the involvement of bacteria.^2^ Erosion has a multifactorial etiology and is related to intrinsic and extrinsic factors. Intrinsic factors are related to endogenous acids produced by the human body and commonly present in individuals with bulimia or diseases affecting the gastrointestinal tract.^3^ The extrinsic factors are related to exogenous acids found in foods and beverages.^2^

Several commercially available acidic beverages accelerate the erosion process, such as citric acid-based^4^ ^-^ ^8^ and cola-based^3^ ^,^ ^4^ ^,^ ^7^ ^-^ ^14^ drinks, energy drinks,^15^ and isotonic drinks.^4^ The erosive potential of these beverages is related to their low pH and low buffering capacity. Acidic foods and beverages with pH lower than 5.5 may cause the dissolution of hydroxyapatite and fluorapatite present in tooth enamel.^5^

Tooth enamel is a mineralized tissue and its microstructure influences the bonding mechanism involving this substrate and the bracket.^1^ A satisfactory bond between bracket and enamel is crucial for the success of the orthodontic treatment, considering that the bonded bracket, apart from the fact that it will eventually be removed, should resist the orthodontic forces and the masticatory loads occurring during the treatment.^16^ Oncag et al^13^ found that carbonated beverages, such as Coca-Cola™ and Sprite™, negatively affected the retention force of brackets bonded to enamel previously subjected to an erosion process. On the other hand, Khoda et al^14^ showed that the intake of acidic beverages does not decrease the bond strength of orthodontic brackets to tooth enamel.

Some behavioral factors such as eating habits may change bracket bond strength to enamel during orthodontic treatment. The pH of beverages, type of acid present, buffering capacity of saliva, constant acidity (pKa), and concentrations of phosphate, calcium, fluoride and phosphorus may influence the erosion of hard dental tissues.^3,10-12^ Few current studies correlate the bonding of orthodontic attachments in previously eroded enamel and its potential complications.^17^

This study aimed to assess the effect of storage time in the erosive solution and the effect of the substance used in the erosive challenge on the bond strength of metal orthodontic brackets bonded to bovine enamel. The null hypotheses were: [1] there would be no differences on the bond strength of the brackets to enamel due to the different immersion times, and [2] the different solutions would not affect the bond strength of the brackets to enamel.

## MATERIAL AND METHODS

This *in vitro* study was carried out with 60 bovine central incisors. Based on the study by Pasha et al,^18^ who found the highest standard deviation of the groups equal to 2.74 MPa, at 5% significance level, in order to prove that 7 elements per group are required to detect a minimum difference of 2.5 MPa among groups. Predicting potential losses, the number of 10 elements^13^ ^,^ ^18^ per group was adopted. These teeth were sectioned in sizes of 7*x*7*x*2 mm, at the flattest central region of the buccal aspect in the cervical-incisal and mesiodistal directions, forming enamel blocks,^7^ and poured in acrylic resin within a polyvinyl chloride (PVC) tube. The same evaluator performed all the procedures.

**Figure 1:**
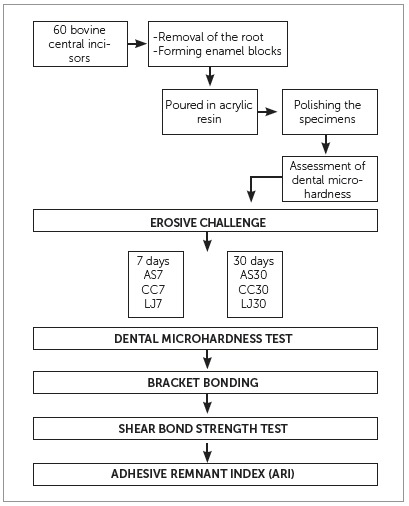
- Flowchart of the method design of the study.

The random distribution of specimens in their respective groups was performed as follows: specimens were numbered from 1 to 60, placed in one single recipient, and picked one by one to compose the groups. The groups were separated by time (7 and 30 days) and by the beverage used (Coca-Cola [CC], Lime Juice [LJ] and artificial saliva [AS]), arranged as follows: AS7; CC7; LJ7; AS30; CC30; LJ30 (Fig 1).

The surfaces were flattened and polished in order to standardize the specimens and prepare them for the dental microhardness test. Therefore, #320, #600, and #1200 silicon carbide grit papers^9^ ^,^ ^12^ ^,^ ^19^ ^-^ ^21^ (Norton™, Guarulhos/SP, Brazil) were used for 30 seconds in high rotation and refrigeration in the polisher (Politriz Polipan™ 2, São Paulo/SP, Brazil).

### Assessment of dental microhardness testing

**Figure 2:**
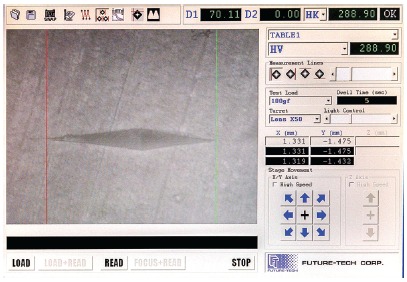
- Indentation performed by Knoop microdurometer. Red and green lines delimiting the indentation size.

In order to verify the standardization of enamel surface hardness^19^ a microhardness tester (FM 700, Future Tech Corp., Tokyo, Japan) was used, as well as a Knoop indenter with 100 g of static load, for 5 seconds on enamel (Fig 2). Three indentations were made on the same specimen according to the following protocol^19^: one indentation to the right, one in the middle, and one to the left, with distance of 100 µm separating each indentation.^5^ To conclude the test, samples were subjected to the erosive process with the selected beverages.

### Measurement of pH 

The pH was measured in a previously calibrated bench pH meter (Q400AS Quimis™, Diadema/SP, Brazil). Thirty mL of each compound were placed in a test tube and tested in the glass electrode of the pH meter, and the value obtained was shown in the ATT digital display.^4^ The operation was repeated three times with a five-minute interval, to standardize and certify the values obtained in the test (Table 1).

**Table 1:** Description of solutions composition and their pH value.

Immersion solution	Composition	pH value
Artificial saliva	Ca(NO_3_)_2_; H_2_O 1.5 mmol/L Na_2_HPO_4_; 2H_2_O 0.9 mmol/L KCI 150 mmol/L H_2_NC(CH_2_OH)_3_ (TRIS) 0,1 mol/L NaF 0,05 µg/mL	6.5
Coca-Cola^TM^	Carbonated water, sugar, kola nut extract, caffeine, caramel coloring IV, acidulant INS 338 and natural aroma	2.32
Natural One™ lime juice	Lime juice without preservatives, with sugar	2.77

### Immersion method

Immersion cycles were performed by submerging specimens in the specific solution for five minutes,^4^ four times a day (8h, 12h, 16h, and 20h),^20^ under agitation, for seven^19^ and 30 days.^4^ The solutions composition is described in Table 1. After each immersion cycle, the specimens were washed in distilled water, dried in absorbent paper, and immersed in 15 mL of artificial saliva; then they were incubated at 37^o^C until the immersion procedure.^4^ In the AS7 and AS30 groups, specimens were immersed in artificial saliva for the selected time. Saliva was changed weekly^4^ for groups AS30, CC30, and LJ30, due to the longer testing period. After all immersions were concluded, the specimens were kept in distilled water at room temperature.

### Bonding procedure

The metal orthodontic brackets, Roth prescription, with 0.022-in slot (3M Unitek, São José do Rio Preto/SP, Brazil), were bonded to bovine tooth surfaces with Transbond™ XT orthodontic adhesive system (3M Unitek, Monrovia, CA, USA). Prophylaxis was previously performed with an extra-thin pumice (S.S. White, Rio de Janeiro/RJ, Brazil) and distilled water solution with a Robinson brush (Microdont, São Paulo/SP, Brazil) for 10 seconds in low-rotation handpiece (Kavo, Joinville/SC, Brazil), and water sprayed (manufacturer’s recommendation). Acid-etching was performed with 37% phosphoric acid (Dentsply, Petrópolis/RJ, Brazil) for 30 seconds on the dental surface, followed by water spraying and air-drying. Next, a primer was applied to the etched sample according to the manufacturer’s protocol and light-cured for 15 seconds.

The adhesive was applied with a syringe (from the Transbond™ XT kit) using a sufficient amount to completely fill the base of the bracket. Then, the bracket was lightly placed on the dental surface aided by orthodontic tweezers, and pressed to remove excesses. The structure composed by tooth/adhesive system/bracket was light-cured for 20 seconds.

### Shear bond strength test

Specimens were subjected to the shear bond strength test using a universal testing machine (EMIC DL-1000, São José dos Pinhais/PR, Brazil) with 10 KN maximum capacity, 50 KgF cell load^21^ and 0.5 mm/min crosshead speed,^5^ 48 hours^22^ ^-^ ^23^ after bonding the orthodontic attachments.

**Figure 3:**
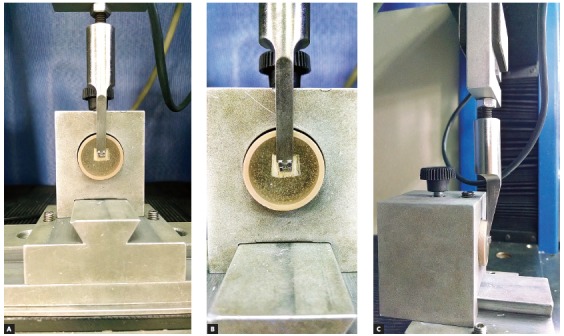
Shear test at EMIC: A) front view; B) position of the chisel tip on the upper surface of the bracket; C) side view performing the test.

Specimens were positioned in the testing machine so that the vertical rod of the shearing machine was perpendicular to the incisal edge of the bracket (flattest part), close to enamel surface, and parallel to the latter (Fig 3), in such a way that the force was perpendicular to the orthodontic bracket during the test.^5^ The force required for detachment was obtained in Kilograms-force (KgF), then converted into Newtons (N), and finally recorded and divided by the bonding area (area of the base of the bracket = 12.89 mm^2^), thus obtaining bond strength values in MegaPascal (MPa).

### Adhesive Remnant Index (ARI)

After shear test, the Adhesive Remnant Index (ARI) was assessed with a stereoscope (SteREO Discovery. V20, Zeiss, Germany) with 10*x* magnification.

Any adhesive remaining after bracket removal was assessed according to the ARI.^24^ The ARI scale ranges from 5 to 1, where 5 indicates that no composite remained on the enamel; 4 = less than 10% of the composite remained on the tooth surface; 3 = between 10% and 90% of the composite remaining; 2 = more than 90% remained on the tooth, and 1 = all composite remained on the tooth, along with the impression of the bracket base.

### Statistical analysis

The Kolmogorov-Smirnov test was used to verify sample normality. Two-way ANOVA (solution factor and treatment time factor) was used for statistical analysis of data, followed by Tukey’s *post-hoc* and Student’s *t* tests for paired samples. All analyses considered a significance level of 95% and all tests were performed in the SPSS 16.0 software (IBM).

The Kruskal-Wallis non-parametric test was used to compare the six groups, regarding the ARI score.

All statistical procedures were performed in the Statistica software (StatSoft Inc., Tulsa, USA) version 13.

## RESULTS

The sample showed normal distribution according to the Kolmogorov-Smirnov test (p = 0.77). The mean value (±standard deviation) of enamel microhardness was 281.89 ± 44.51 KHN.

**Figure 4:**
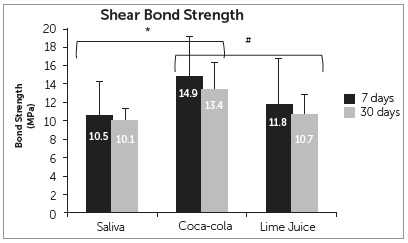
Effect of the type of solution and immersion time on shear bond strength measurements. The values in the bars refer to the mean and standard deviation. The * and the # represent statistically significant difference for the “solution” factor (F_5.54_= 6.671; p_ _= 0.003) among solutions of Saliva x Coca-Cola™ (p_ _= 0.003) and Coca-Cola™ x Lime Juice (p_ _= 0.029).

Two-way ANOVA followed by Tukey’s test showed significant effect only for the solution factor (F_5.54_= 6.671; *p*_ _= 0.003), while the immersion time factor (F_5.54_= 1.282; *p*_ _= 0.263) and the interaction among the factors studied were not statistically significant (F_5.54_= 0.105; p_ _= 0.901). Figure 4 shows that Tukey’s test identified a statistical difference among the bond strength values of Artificial Saliva *versus* Coca-Cola™ (p_ _= 0.003) and Coca-Cola™ *versus* Lime Juice (p_ _= 0.029), regardless of the immersion time. The bond strength values obtained for the group immersed in Coca-Cola™ were significantly higher when compared to those of the groups subjected to Artificial Saliva and Lime Juice.

Table 2 lists the ARI scores. In the 7-days protocol, both in Saliva and Lime Juice groups, 40% of samples presented all the adhesive on the enamel surface. On the other hand, in the Coca-Cola™ group, 40% of samples indicated score 4, that is, less than 10% of adhesive on the enamel surface. In the 30-days protocol, in the Saliva group, 40% of samples indicated score 4, that is, less than 10% of adhesive on the enamel surface. In the Lime Juice group, scores 1 (all adhesive on teeth) and 3 (more than 10% and less than 90% of adhesive on the enamel surface) were the most recurrent scores. In the Coca-Cola™ group, score 5 was mostly repeated, indicating no adhesive on the enamel surface.

**Table 2:** Rate of occurrences of ARI score, median score, and result of the comparison between the groups by the Kruskal-Wallis test.

Time	Solution	ARI score*					Median	p
		1	2	3	4	5	score	
7 days	Saliva	40%	20%	10%	20%	10%	2	0.475
	Coca-Cola	20%	10%	30%	40%	0%	3	
	Lime juice	40%	30%	10%	20%	0%	2	
30 days	Saliva	30%	10%	20%	40%	0%	3	
	Coca-Cola	30%	0%	20%	0%	50%	4	
	Lime juice	30%	0%	30%	20%	20%	3	

*1 = all composite remained on tooth, 2 = more than 90% of composite remained on tooth, 3 = between 10% and 90% remained on tooth, 4 = less than 10% of composite on tooth, 5 = no composite on tooth.

## DISCUSSION

The results of this study indicated that the storage in the erosive solutions (Coca-Cola™ and lime juice) did not affect the bond strength of the brackets to bovine enamel. However, the type of erosive solution had a significant effect on the bond strength, whereas immersion in Coca-Cola™ resulted in significantly higher mean values than those obtained after immersion in lime juice. Thus, the first null hypothesis was accepted and the second one was rejected. 

There is a diversity of protocols of *in vitro* erosive challenges that range from three days^10^ to three months^14^ regarding immersion time; and from two^13^ to four^16^ times a day, regarding the number of immersions; there is also a great variation of types of food and beverages investigated. The literature shows a higher number of researches using Coca-Cola™,^3^ ^,^ ^4^ ^,^ ^7^ ^-^ ^14^ followed by critic beverages^4^ ^-^ ^8^ ^,^ ^14^ such as lime-flavored soft drinks or lime juice. This research used Coca-Cola™ and Natural One™ lime juice. Both have an acid pH (Coca-Cola™= 2.32, Lime Juice= 2.77), favoring tooth enamel dissolution, which enables researches that induce the *in vitro* erosive challenge.^2^

Oncag et al^13^ induced erosion both *in vivo* and *in vitro*. The protocol adopted was the immersion for 5 minutes in predefined substances (Coca-Cola™ and Sprite™) and the control (artificial saliva), three times a day for three months. They noticed that there was no statistically significant difference between *in vivo* and *in vitro* groups. Khoda et al,^14^ tested only *in vitro* using a similar protocol (immersion for 5 minutes three times a day for three months); however, with drinks of similar brands (Pepsi™ and 7Up Soda™). In the first work, the erosion caused by Coca-Cola™ and Sprite™ decreased the shear strength of the bracket to the enamel both *in vivo* and *in vitro*. In the second study, as a result, they observed that there was no negative effect on the shear strength in the bracket-enamel relationship. In addition to the difference in the drinks, the cementing agent may interfere with the final results as well as the time when the enamel was eroded before or after the bracket bonding. In works that simulated what would happen *in vivo,* there is also no homogeneity among protocols. Kato and Buzalaf^12^ used an *in situ* protocol by means of removable apparatus adapted with blocks of enamel, in which the individual removes the apparatus and immerses it in the substance in the predetermined time (immersion for 5 minutes, four times a day, for 5 days); and also observed wear on the surface of the enamel. In general, the difference among protocols hinders and prevents a more reliable comparison among results. In *in vivo* protocols it becomes even more difficult to discuss, due to the reduced number of papers and concerning with the ethical precepts.

In this research, two erosive challenge protocols were tested before bracket bonding. Immersion of the *in vitro* specimens was performed four times daily, for 5 minutes, over 7 and 30 days. It was observed that time does not influence the type of protocol. However, the beverages used during the experiment have a direct influence on the final result, as noted in other studies.^13-14^

There were statistically significant differences only for Coca-Cola™ in relation to the other solutions (Lime Juice and Saliva) in both 7 and 30 days, but when these storage time protocols (7 and 30 days) were compared within the same solution, it was not observed statistically significant difference. When substance pH is lower than 4, as were the tested substances, saliva tends to become sub-saturated in hydroxyapatite and fluorapatite, limiting its remineralizing action and justifying the absence of complete remineralization on dental surfaces subjected to the erosive challenge.^6^ ^,^ ^24^ Fushida and Cury^26^ assessed the erosive effect of Coca-Cola™ on enamel and dentin and found no complete remineralization as well. 

The statistically significant difference found for Coca-Cola™ in relation to lime juice in both storage times (7 and 30 days) may be justified by the different acids present in the composition of beverages. A study^25^ showed that the phosphoric acid present in Coca-Cola™ has higher erosive potential than the citric acid present in lime juice. Besides the different acids, factors such as pH, mineral content, titratable acidity, and chelation properties of calcium may change the erosive potential of both beverages.^2^

The acids in Coca-Cola™ and lime juice lead to demineralization of dental inorganic matrix.^4^ ^,^ ^25^ ^,^ ^27^ The longer the exposure time to etiological factor, the greater the lesion size.^24^ However, the stability of enamel hydroxyapatite crystals in an erosive challenge may be maintained when phosphate, calcium, and/or fluoride ions are added.^3^ ^,^ ^10^ ^-^ ^12^ In this research, such ions weren’t added to the tested substances. 

Previous studies^5^ ^,^ ^13^ ^,^ ^25^ showed that when the erosive challenge was performed after bracket bonding, the shear bond strength decreased relative to the control group. This result may be justified by the degradation of the adhesive system around the attachment, in the bracket/adhesive system/tooth junction, stimulated by the acids present in the beverages.^5^ ^,^ ^13^ ^,^ ^16^ This research used a different method from the one previously mentioned, because the erosive challenge process was performed before orthodontic attachment bonding, simulating the erosive wear on enamel from the habit of drinking acidic beverages prior to the orthodontic treatment. 

Reynolds^28^ affirmed that a value of 4.9 MPa seems reasonable for clinical success in order to maintain brackets bonded, considering they should bear this level of masticatory and orthodontic stresses without detachment. Sheibaninia et al^29^ found values ranging from 11 to 27 MPa for shear bond strength, but without fractures. In the present research, the bond strength values found, after 7 and 30 days, were higher for groups immersed in Coca-Cola™ (14.9 and 13.4 MPa) and lime juice (11.8 and 10.7 MPa) than the values obtained for the control group with artificial saliva (10.5 and 10.1 MPa). Pasha et al^18^ showed that the erosive challenge with Coca-Cola™ presents higher shear strength and greater superficial wear on enamel than other substances. Barac et al^30^ assessed enamel roughness after immersion in five beverages, including Coca-Cola™, which presented a higher erosion potential and resulted in higher superficial roughness on enamel than the other substances. It is suggested that roughness may be one of the factors that influence enamel/adhesive system interlocking, possibly inducing higher shear bond strength. 

The Adhesive Remnant Index was used to assess the pattern of adhesive failure, and there was no statistically significant difference between groups, as reported by Baka et al.^31^ Sajadi et al.^16^ obtained the same result, but identified higher tendency of adhesive to remain on the bracket mesh rather than on enamel, as in the present study. This suggests a higher connection between orthodontic bracket and adhesive than between adhesive and enamel. Both Sajadi et al.^16^ and Baka et al.^31^ showed in their results that the most recurrent ARI was the one where the adhesive remained in full or almost completely on the orthodontic attachment mesh. Sheibaninia et al^29^ affirm that, when this pattern occurs, it is for lacking a connection between adhesive system and tooth enamel. 

This investigation showed that teeth subjected to constant erosive induction and requiring posterior bracket bonding may suffer higher resistance when removing orthodontic attachments. However, these *in vitro* results may not be extrapolated for *in vivo* conditions. Clinical studies should be conducted, substantiating the laboratory results.

## CONCLUSIONS

The immersion time used in the erosion protocols did not affect the bond strength of brackets to teeth after 7 and 30 days of *in vitro* erosive challenges. Regarding the influence of the acidic beverages on the adhesion of orthodontic brackets, the immersion of bovine teeth in Coca-Cola™ induced significantly higher shear bond strength values than the immersion in lime juice and artificial saliva.
